# Headaches during/after SARS‐CoV‐2 infection/vaccination can be primary and secondary as well as acute and chronic

**DOI:** 10.1111/ene.16307

**Published:** 2024-04-15

**Authors:** Josef Finsterer, Sounira Mehri

**Affiliations:** ^1^ Neurology and Neurophysiology Center Vienna Austria; ^2^ Biochemistry Laboratory LR12ES05 ‘Nutrition‐Functional Foods and Vascular Health’, Faculty of Medicine Monastir Tunisia

We read with interest Mitsikostas et al.'s [1] systematic review of facial pain and headaches in patients with severe acute respiratory syndrome coronavirus 2 (SARS‐CoV‐2) infection (SC2I) or after SARS‐CoV‐2 vaccination (SC2V). No data on SC2I/SC2V‐related facial pain were identified [1]. SC2I‐related headaches were reported in 6.5%–74.6% of cases and have been attributed to immune‐mediated mechanisms that activate the trigeminovascular system [1]. The prevalence of headache differed significantly between prospective and retrospective studies [1]. SC2V‐related headaches occurred in 13%–77% of vaccinees, more often in females and those receiving vector vaccines [1]. The study is excellent, but some points need discussion.

The first point is that no distinction has been made between headache/facial pain in the acute stage of the disease and long COVID syndrome or between acute COVID vaccination syndrome (ACVS) and post‐acute COVID vaccination syndrome (PACVS) [2]. Headache cannot only occur in the acute stage of infection but can also be a feature of long COVID syndrome [3]. In a study of 114 patients with long COVID syndrome, 22.6% reported persistent headaches [3]. Likewise, 7.3% of 137 frail nursing home residents reported headaches 6 months after primary vaccination [4]. Headaches in long COVID and PACVS may have a different character, treatment need and response, and outcome than headaches in acute SC2I or ACVS.

A second point is that the absence of facial pain as a complication of SC2I or SC2V is incomprehensible. There are several reports about trigeminal neuralgia following SC2I or SC2V [5]. New‐onset trigeminal neuralgia after SC2V has been reported, particularly in patients with a history of rheumatological disease. There are also some reports of SC2V‐induced arteritis temporalis. Another cause of facial pain may be orbital myositis, which has been described as a rare complication of SC2I and SC2V.

A third point is that the classification of SC2I/SC2V‐associated headaches as only primary headaches is incomprehensible. SC2I/SC2V‐associated headaches can also be secondary, as they are triggered by complications of the SC2I or SC2V.

The fourth point is that several causes of secondary headache in patients with SC2I/SC2V have not been considered. For example, central nervous system (CNS) vasculitis following SC2I was not considered a cause of headache. CNS vasculitis has been reported not only in adults but also in children. SC2V‐associated CNS vasculitis can be complicated by stenosis or even occlusion of cerebral arteries. Another overlooked form of SC2I/SC2V‐associated headache is reversible cerebral vasoconstriction syndrome. Although it is rarely reported, it should not be neglected as a secondary type of SC2I/SC2V‐associated headache because it is most likely underreported. This is because many headache patients do not receive appropriate imaging and, in particular, information about the arteries is often not recorded.

The fifth point is that venous sinus thrombosis (VST) after SC2I or SC2V is not rare, as stated in the review [1]. In a study of 910,556 patients who received the first dose of an SC2V and 165,863 patients with SC2I, 1372 VST events were recorded.

In summary, the excellent study has limitations that should be addressed before final conclusions are drawn. SC2I/SC2V can be complicated not only by headaches but also by facial pain. Headaches can occur in the acute and chronic stages of SC2I as well as in ACVS and PACVS. Headaches after SC2I/SC2V can be primary and secondary.

## AUTHOR CONTRIBUTIONS

Josef Finsterer: validation; investigation; project administration; data curation; writing—review and editing. Sounira Mehri: investigation; formal analysis; supervision; project administration.

## CONFLICT OF INTEREST STATEMENT

The authors declare that the research was conducted in the absence of any commercial or financial relationships that could be construed as a potential conflict of interest.

## ETHICS STATEMENT

The study was approved by the institutional review board (responsible: Finsterer J.) on 4 February 2024. This article is based on previously conducted studies and does not contain any new studies with human participants or animals performed by any of the authors.

## INFORMED CONSENT

Written informed consent was obtained from the patient for publication of the details of their medical case and any accompanying images.

## Data Availability

Data that support the findings of the study are available from the corresponding author.
